# Lack of association between single-nucleotide polymorphisms of pro- and anti-inflammatory cytokines and HTLV-1-associated myelopathy / tropical spastic paraparesis development in patients from Rio de Janeiro, Brazil

**DOI:** 10.1186/s12879-018-3510-1

**Published:** 2018-11-22

**Authors:** Doris Schor, Luís Cristóvão Porto, Eric Henrique Roma, Marcel de Souza Borges Quintana, Gustavo Milson Fabricio-Silva, Maria Gloria Bonecini-Almeida, Abelardo Queiroz-Campos Araújo, Maria Jose Andrada-Serpa

**Affiliations:** 1 Laboratory of Immunology and Immunogenetics in Infectious Diseases, Evandro Chagas National Institute of Infectious Diseases, FIOCRUZ, Avenida Brasil, 4365, Manguinhos, Rio de Janeiro, RJ 21040-360 Brazil; 2 Laboratory for Clinical Research in Neuroinfections, Evandro Chagas National Institute of Infectious Diseases, FIOCRUZ, Rio de Janeiro, RJ Brazil; 3 Histocompatibility and Cryopreservation Laboratory, Policlinica Piquet Carneiro, Rio de Janeiro State University (UERJ), Rio de Janeiro, RJ Brazil; 4 Clinical Research Platform, Evandro Chagas National Institute of Infectious Diseases, FIOCRUZ, Rio de Janeiro, RJ Brazil

**Keywords:** Cytokine, HAM/TSP, HTLV-1, SNP, Proviral load

## Abstract

**Background:**

HTLV-1-associated myelopathy/tropical spastic paraparesis (HAM/TSP) is a progressive neurological and inflammatory disease, associated with HTLV-1 infection. HAM/TSP neurological disease is a consequence of an inflammatory reaction, and adaptive immune responses, through the secretion of anti-inflammatory and pro-inflammatory cytokines, play an important role in the outcome of infection and disease progression. Studies addressing the association between cytokines functional single nucleotide polymorphisms and HAM/TSP development are scarce.

**Methods:**

The genetic polymorphisms of cytokine genes were evaluated in HAM/TSP patients (*n* = 68) and in asymptomatic HTLV-1 positive carriers (*n* = 83) from Rio de Janeiro, Brazil, in a case-control study. HTLV-1 infected patients were genotyped for SNPs in five cytokine genes: *TNFA-308G/A, IL6-174G/C, IFNG + 874 T/A, TGFB* at the codons *+ 10 T/C and* + *25G/C*, *IL10-592C/A and -819C/T, and -1082A/G* and proviral load (PVL) was quantified. Associations between genotypes, haplotypes, clinical outcome and pro viral load were evaluated.

**Results:**

Lack of association between the cytokine polymorphisms and disease outcome was observed. The genotypes *TNFA-308GG, IL6-174GG/GC, IL10-592AA* and *-819CC* and TGFb1 high producers phenotypes were correlated with higher PVL in HAM/TSP patients versus asymptomatic carriers.

**Conclusions:**

We did not observe association between cytokine polymorphisms and risk for HAM/TSP development in Brazilian HTLV-1 infected individuals, regardless of differences in PVL between HAM/TSP versus asymptomatic carriers in specific cytokine polymorphisms.

## Background

The HTLV-1 virus is the etiological agent of two major diseases: adult T cell leukemia and the neurological disease HTLV-associated myelopathy / tropical spastic paraparesis (HAM/TSP), a progressive neurological and inflammatory disease of the central nervous system. Although many of the infected individuals are asymptomatic, approximately 2–5% of the infected individuals will develop HAM/TSP [1]. This neurological disease could be the consequence of an inflammatory network that results in damage of the spinal cord [2], but this association is still poorly understood.

Tax viral protein plays an important role in the regulation of the virus genome acting in proviral genome transcription by interacting with several cellular signaling pathways that modulate the expression of cytokine and chemokine genes [3]. The IFN-γ secreted by HTLV-1 infected CD4^+^T cells and the virus recognition by CD8^+^T lymphocytes in the central nervous system induces production of other cytokines, such as the myelinotoxic TNF-α. This event, together with the hemodynamic changes and interactions mediated by adhesion molecules among circulating lymphocytes and endothelial cells, which contribute to the location of spinal cord injury, is known as a “bystander” damage hypothesis. The involvement of cytokines in the outcome of HAM/TSP is associated with the “bystander” damage hypothesis [1]. Among the potential immunopathological findings, high levels of IFN-γ, TNF-α and IL-6 have been detected in patients with HAM/TSP, which seem to contribute for the breakthrough of the blood–brain barrier and resulting in immunopathology and neurological symptoms [4]. In addition, HAM/TSP patients showed dysregulation in TGF-β signaling, affecting Treg function and contributing for disease pathology [5]. Differences in IL-2, IL-4, IL-10, IL-12p70, TNF-α and IFN-γ levels were also found in the supernatants of cultured peripheral blood mononuclear cells (PBMC) from HAM/TSP and AC [6]. Recent publication showed no difference in plasma cytokine levels among AC and HAM/TSP patients. However, cerebrospinal fluid levels of cytokines (ITAC, IFN-γ, IL-5, IL-8 and TNF-α) were higher in HAM/TSP compared with AC patients, indicating that those cytokines might be used as disease markers of neurologic manifestation in long-term HTLV-1 infected individuals [7].

Single nucleotide polymorphisms (SNPs) might have influence in the cytokine production. It has been shown that *IFNG + 874 T/A* [8], *TNF -308G/A* [9, 10], *IL6 -174G/C* [11], *IL10–1082A/G*, *-819C/T* and *-592C/A* [12], and *TGFB* at the codons *+ 10 T/C* and *+ 25G/C* [13, 14] SNPs alter the expression of the related cytokines in pathological or physiological conditions. Many of these polymorphisms have been associated with inflammatory and infectious diseases [15–19]. Indeed, *IFNG + 874A/T* [20], *IL6 -174G/C* [21], and *IL10 -592C/A* and -819C/T [22] have been associated with HAM/TSP development or HTLV-1 infection.

Because these specific polymorphisms are associated with changes in cytokines production and many inflammatory and infectious diseases, and those cytokines have important role in HTLV-1 infection, it would be important evaluate the association between these polymorphisms and HAM/TSP development. Studies addressing the role of SNPs that might alter cytokine production and HTLV-1 infection are scarce. Therefore, our aim was to determine in a case-control study if SNPs of pro- and anti-inflammatory cytokines in HAM/TSP and AC patients were related to disease outcome.

## Methods

### Study population

HAM/TSP patients and ACs were randomly recruited from a cohort of approximately 700 individuals attended at the Laboratory for Clinical Research in Neuroinfection, INI-FIOCRUZ, Rio de Janeiro, RJ, Brazil. The diagnosis of HAM/TSP patients was performed according to the World Health Organization diagnostic criteria [23]. The patients were mainly from Rio de Janeiro, Brazil, and the follow-up mean was 128.85 ± 54.44 months. The Institutional Ethics Committee approved the study, and all subjects provided written informed consent. Demographic data included information regarding a self-identified skin color as described as white or non-white (black and mixed persons), sex and age.

### DNA extraction

Five milliliters of whole blood were collected in EDTA-containing tubes, and DNA extraction was carried out by a commercial kit from Puregene (Gentra Systems Inc., Minneapolis, MN, USA), following the manufacturer’s instructions.

### Genotyping

SNPs in five cytokine genes, *TNFA-308G/A, TGFB* at codons *+ 10 T/C and TGFB* + *25G/C*, *IL10-1082A/G, -592C/A and -819C/T, IL6-174G/C, IFNG + 874 T/A* were genotyped through sequence-specific primers-polymerase chain reaction technique (SSP-PCR) using a commercial kit by Cytokine Genotyping Tray (One Lambda, Inc., Canoga Park, CA, USA). This technique has allowed us genotyped eight SNPs of five cytokine genes, simultaneously, through PCR reaction. The SSP-PCR technique provides an accurate, simple, and economical means of detecting polymorphisms of these important pro- and anti-inflammatory cytokine genes.

Ninety-six well microtiter® trays with dried primers in each reaction well were filled with 10 μl of a mix containing dNTPs (8 μl, provided by the manufacturer), Taq polymerase (1 μl at 5 U/μl) and DNA sample (1 μl, 100 ng) per reaction well. The reaction was performed in a Veriti thermocycler (Applied Biosystems, Foster City, CA, USA) with the follow instructions: one cycle of 130 s at 96 °C, 60s at 63 °C; nine cycles of 10s at 96 °C, 60s at 63 °C; twenty cycles of 10s at 96 °C, 50s at 59 °C and 20s at 72 °C; and ending at 4 °C.

After completing the PCR reaction, the samples were transferred to 2.5% agarose gel stained with ethidium bromide (0.5 μg/ml), and were electrophoresed at 140–150 V. After electrophoretic run, the gel was transferred for an ultraviolet transilluminator and photographed for analysis. The presence or absence of gel band from each amplified reaction well determined the SNP identification.

Phenotype analysis for *IL10-1082A/G, -819C/T and -592C/A* SNPs were demonstrated as low (ACC/ACC, ACC/ATA, ATA/ATA), intermediate (GCC/ACC, GCC/ATA) and high (GCC/GCC) IL-10 producers, as described by the commercial kit.

### Proviral load quantification

HTLV-1 PVL DNA from peripheral blood leucocytes was measured by real-time PCR assay (SmartCycle II; Cepheid) using the TaqMan system (Applied Biosystems, Foster City, California, USA), through the amplification of a 159-bp fragment of the *tax* gene. As a reference, a standard curve was generated using the human *β-globin* gene and DNA from the TARL-2 cell line, which contains a single copy of the provirus HTLV-1, to establish the *tax* gene quantification. PVL was calculated in peripheral blood leukocytes (PBL) from each volunteer following the equation: [(copy number of *tax* gene) / (copy number of β-globin gene/2)] × 100 [24].

### Statistical analysis

Proviral load statistical analysis between genotypes/phenotypes and disease groups was performed by an ordinary two-way ANOVA with Bonferroni post-test for multiple comparisons correction [25], considering results with *p-*values < 0.05 statistically significant. Nonparametric data were evaluated by Kruskal-Wallis and Mann-Whitney tests and a Spearman correlation, and association between qualitative variables (alleles, genotypes and phenotypes frequencies) was tested by chi-square, Cochran-Armitage or Fisher’s exact test. *P*-values from tables with any significant result were corrected using Bonferroni correction for multiple comparisons. Results with a *p*-value < 0.05 were considered statistically significant. All analyses were performed using the software GraphPad Prism 6.0 or R version 3.1.0 [26].

## Results

### Characteristics of HTLV-1 infected patients and proviral load

Sixty-eight HAM/TSP patients and 83 ACs were enrolled. The demographic data are shown in Table 1. We did not observe differences related to ethnic background, gender and age between the grouped volunteers. Proviral load was higher in the HAM/TSP patients than AC group(*p* < 0.04, Table 1).

**Table 1:** Demographic variables distribution according with clinical condition of HAM/TSP patients

Variables	HAM/TSP	Asymptomatic	OR (CI:95%)	*p*-value	Corrected *p*-value^d^
Skin color (n/%)					
White	40 (46.5)	46 (53.5)	1		
Non-White	28 (43.1)	37 (56.9)	0.87 (0.45–1.66)	0.67^a^	1.00
Sex (n/%)					
Male	27 (41.5)	38 (58.5)	1		
Female	41 (47.7)	45 (52.3)	1.28 (0.67–2.47)	0.45^a^	1.00
Age					
Mean (SD)	57.8 (11.3)	53.3 (14.7)	1.03 (1–1.05)	0.04^b^	0.16
PVL Median (IQR)	7.1(4.0–9.8)	1.3 (0–4.9)	1.14 (1.06–1.24)	< 0.01^c^	< 0.04

*HAM/TSP* HTLV-1-associated myelopathy / Tropical spastic paraparesis, *OR* odds ratio, *CI* confidence interval, *SD* standard deviation, *PVL* proviral loads

^a^Chi-Square or Fisher Exact test. ^b^T-test for comparison of difference means and ^c^Kruskal-Wallys test. ^d^Adjusted *p*-value using Bonferroni correction for multiple comparisons

### Distribution of cytokines genotypes in HAM/TSP and AC

All analyzed genotype frequencies were in Hardy-Weinberg equilibrium for both the HAM/TSP and AC groups (data not shown). No significant differences were observed at positions *TNFA-308G/A*, *IL6-174G/C*, *IFNG + 874 T/A* and *TGFB* at codons *+ 10 T/C* and *+ 25G/C* between the HAM*/TSP* and AC groups regarding genotypic, allelic and phenotypic analysis in the dominant, recessive or co-dominant genetic models (Tables 2 and 3).

**Table 2:** Analysis of associations between *TNFA-308G/A, IL6-174G/C and IFNG + 874 T/A* polymorphisms and risk of HAM/TSP development

Polymorphisms	HAM/TSP	Asymptomatic		
	*n* (%)	*n* (%)	OR (IC:95%)	*p*-value
*TNFA-308G/A*				
GG	55 (82.1)	69 (83.1)	1	0.98
GA	12 (17.9)	13 (15.7)	1.16 (0.48–2.75)	
AA	0	1 (1.2)	0 (NA)	
GG (low)	55 (82.1)	69 (83.1)	1	1
GA + AA (high)	12 (17.9)	14 (16.9)	1.08 (0.45–2.52)	
AA	0	1 (1.2)	1	1
GA + GG	67 (100)	82 (98.8)	0 (NA)	
*Alleles*				
G	122 (91.0)	151 (91.0)	1	1
A	12 (9.0)	15 (9.0)	0.99 (0.44–2.19)	
*IL6-174G/C*				
GG	48 (71.6)	56 (68.3)	1	0.88
GC	16 (23.9)	24 (29.3)	0.78 (0.37–1.62)	
CC	3(4.5)	2 (2.4)	1.75 (0.28–13.7)	
CC (low)	3 (4.5)	2 (2.4)	1	0.66
GC + GG (high)	64 (95.5)	80 (97.6)	0.53 (0.07–3.31)	
GG	48 (71.6)	56 (68.3)	1	0.79
GC + CC	19 (28.4)	26 (31.7)	0.85 (0.42–172)	
*Alleles*				
G	112(83.6)	136 (82.9)	1	1
C	22 (16.4)	28 (17.1)	0.95 (0.51–1.76)	
*IFNG + 874 T/A*				
AA (low)	34 (50.7)	34 (43.6)	1	0.86
AT (intermediate)	26 (38.8)	40 (51.3)	0.65 (0.33–1.29)	
TT (high)	7(10.4)	4 (5.1)	1.75 (0.48–7.20)	
AA	34 (50.7)	34 (43.6)	1	0.49
AT+TT	33 (49.3)	44 (56.4)	0.75 (0.39–1.44)	
TT	7 (10.4)	4 (5.1)	1	0.35
AT+AA	60 (89.6)	74 (94.9)	0.46 (0.12–1.61)	
*Alleles*				
A	94 (70.1)	108 (69.2)	1	0.97
T	40 (29.9)	48 (30.8)	0.96 (0.58–1.58)	

*HAM/TSP* HTLV-1 associated myelopathy tropical spastic paraparesis, Asymptomatic HTLV-1 patients, *OR* odds ration with confident interval, Chi-square 2 × 2 or 3 × 2 contingent tables, Fisher exact or Cochran-Armitage tests

**Table 3:** Analysis of associations between *TGFB codon 10 T/C* and *codon 25G/C* polymorphisms and risk of HAM/TSP development

Polymorphisms	HAM/TSP	Asymptomatic		
	*n* (%)	*n* (%)	OR (IC:95%)	*p*-value
TGFB Codon 10				
T/T	25 (37.3)	24 (28.9)	1	
T/C	29 (43.3)	45 (54.2)	0.62 (0.3–1.28)	
C/C	13 (19.4)	14 (16.9)	0.89 (0.35–2.29)	0.61
Alleles				
T	79 (59.0)	93 (55.4)	1	
C	55 (41.0)	73 (43.5)	0.89 (0.56–1.41)	0.70
TGFB Codon 25				
G/G	56 (83.6)	71 (85.5)	1	
G/C	10 (14.9)	11 (13.3)	1.15 (0.45–2.92)	
C/C	1 (1.5)	1 (1.2)	1.27 (0.05–32.54)	0.74
T/C G/G High	47 (70.1)	58 (69.9)	1	
T/C G/C; C/C G/G; T/T G/C Intermediate	15 (22.4)	24 (28.9)	0.77 (0.36–1.62)	
C/C G/C; C/C C/C; T/T C/C Low	5 (7.5)	1 (1.2)	6.17 (0.95–120.4)	0.51

*HAM/TSP* HTLV-1 associated myelopathy tropical spastic paraparesis, Asymptomatic HTLV-1 patients, *OR* odds ration with confident interval, Chi-square 2 × 2 or 3 × 2 contingent tables, Fisher exact or Cochran-Armitage tests

The *IL10-1082A/G -592C/A* and *-819C/T* polymorphisms (Table 4) showed the highest frequencies for allele A at position -1082*A/G* and allele C at -819*C/T* and -592*C/A* positions in both groups, with no significant differences in allelic distribution. The polymorphisms *IL10 -592C/A* and *IL10–819C/T* were estimated by a single analysis due to the complete linkage disequilibrium between the two SNPs with D’ = 0.99. The A allele of *IL10 -592C/A* was always linked with the T allele of *IL10–819 T/C* and C with C. No differences were observed regarding genotypic distribution between the HAM/TSP and AC groups in co-dominant genetic models at all positions. Moreover, at position *IL10-1082A/G*, despite we found in a dominant model a 2.4-fold risk factor for HAM/TSP outcome (OR = 2.42 [1.2–4.97], *p* = 0.01) in *IL10-1082A/G* plus *G/G* carriers, the data lost significance due correction for multiple comparisons. Analysis including sex, skin color and age did not interfere with the risk for disease progression (*p* < 0.01, the data not shown). *IL10* phenotypic (low, intermediate and high IL-10 producers) and haplotype (Tables 4 and 5) association showed no influence in HAM/TSP outcome.

**Table 4:** Analysis of associations between *IL10-1082A/G, -819C/*T and *-592C/A* polymorphisms and risk of HAM/TSP development

Polymorphisms	HAM/TSP	AC	Crude		
	*n* (%)	*n* (%)	OR (IC:95%)	*p*-value	Corrected *p*-value
*IL10-1082G/A*					
A/A (low)	20 (32.3)	38 (53.5)	1		
G/A (intermediate)	34 (54.8)	24 (33.8)	2.69 (1.28–5.79)		
G/G (high)	8 (12.9)	9 (12.7)	1.69 (0.56–5.1)	0.07	0.21
A/A	20 (32.3)	38 (53.5)	1		
G/A G/G	42 (67.7)	33 (46.5)	2.42 (1.2–4.97)	0.01	0.07
Alleles					
A	74 (59.7)	100 (70.4)	1		
G	50 (40.3)	42 (29.6)	1.61 (0.97–2.70)	0.07	0.21
*IL10–819/−592* ^a^					
C/C and C/C	25 (43.3)	29 (40.8)	1		
C/T and C/A	31 (50.0)	27 (38.0)	1.33 (0.63–2.8)		
T/T and A/A	6 (9.7)	15 (21.2)	0.46 (0.15–1.33)	0.38	1
C/C and C/C	25 (40.3)	29 (40.8)	1		
C/T + T/T and C/A + A/A	37 (59.7)	42 (59.2)	1.02 (0.51–2.05)	1	1
T/T and A/A	6 (9.7)	15 (21.2)	1		
C/C + C/T and C/C + C/A	56 (90.3)	56 (78.8)	2.5 (0.94–7.44)	0.12	0.84
Phenotype					
ACC/ACC, ACC/ATA, ATA/ATA (Low)	20 (32.3)	38 (53.5)	1		
GCC/ACC, GCC/ATA (Intermediate)	34 (54.8)	24 (33.8)	2.69 (1.28–5.79)		
GCC/GCC (High)	8 (12.9)	9 (12.7)	1.69 (0.56–5.10)	0.07	0.21

*HAM/TSP* HTLV-1 associated myelopathy tropical spastic paraparesis, Asymptomatic HTLV-1 patients, *OR* odds ration with confident interval, Chi-square 2 × 2 or 3 × 2 contingent tables, Fisher exact or Cochran-Armitage tests

^a^-819 and -592 are in linkage disequilibrium

**Table 5:** Haplotype distribution of the *IL-10 (−1082G/A,-819C/T and-592C/A)* according with the clinical condition

Haplotypes	HAM/TSP		Asymptomatic			
	*N*	(%)	*N*	(%)	OR (IC:95%)	*p*-value^a^
GCC	56	(41.18)	54	(32.53)	1	
ATA	49	(29.52)	69	(50.74)	0.64 (0.36–1.12)	
ACC	31	(22.79)	43	(25.9)	0.62 (0.34–1.13)	0.19

*HAM/TSP* HTLV-1 associated myelopathy tropical spastic paraparesis, Asymptomatic HTLV-1 patients, *OR* odds ration with confident interval

^a^Chi-square 3 × 2 contingent tables

### Association with cytokine genotypes and HTLV-1 proviral load

**Fig. 1:**
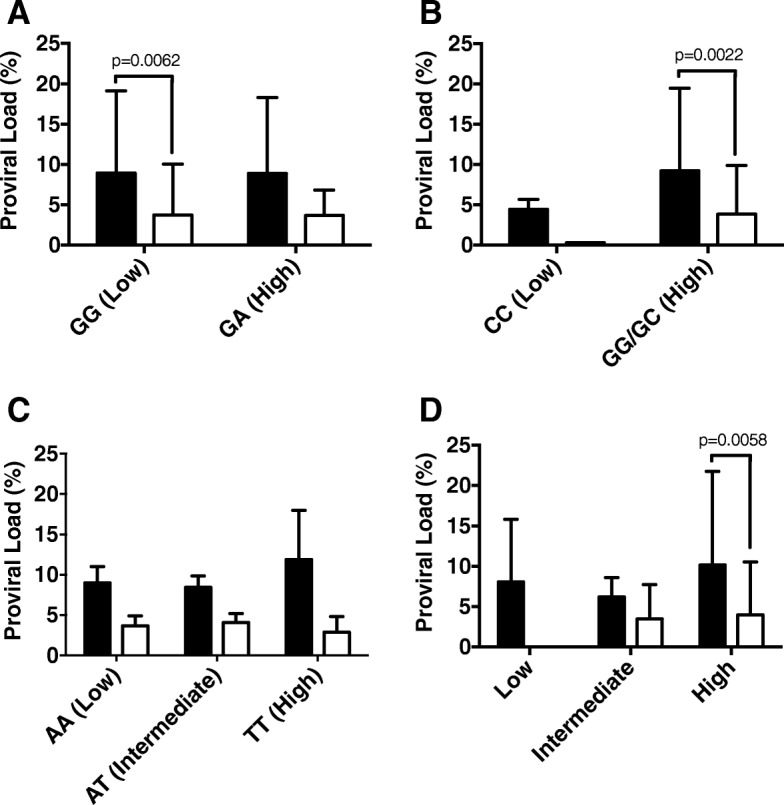
HTLV-1 pro viral load according with cytokines genotypes and phenotypes in HAM/TSP versus AC. The proviral load was expressed as percentage of infected blood leukocytes. **a**, **b** and **c** represent *TNFA-308G/C*, *IL6-174GC* genotypes and **d** represent TGFB1 phenotypes. Statistical significances are indicated according with *p* value

**Fig. 2:**
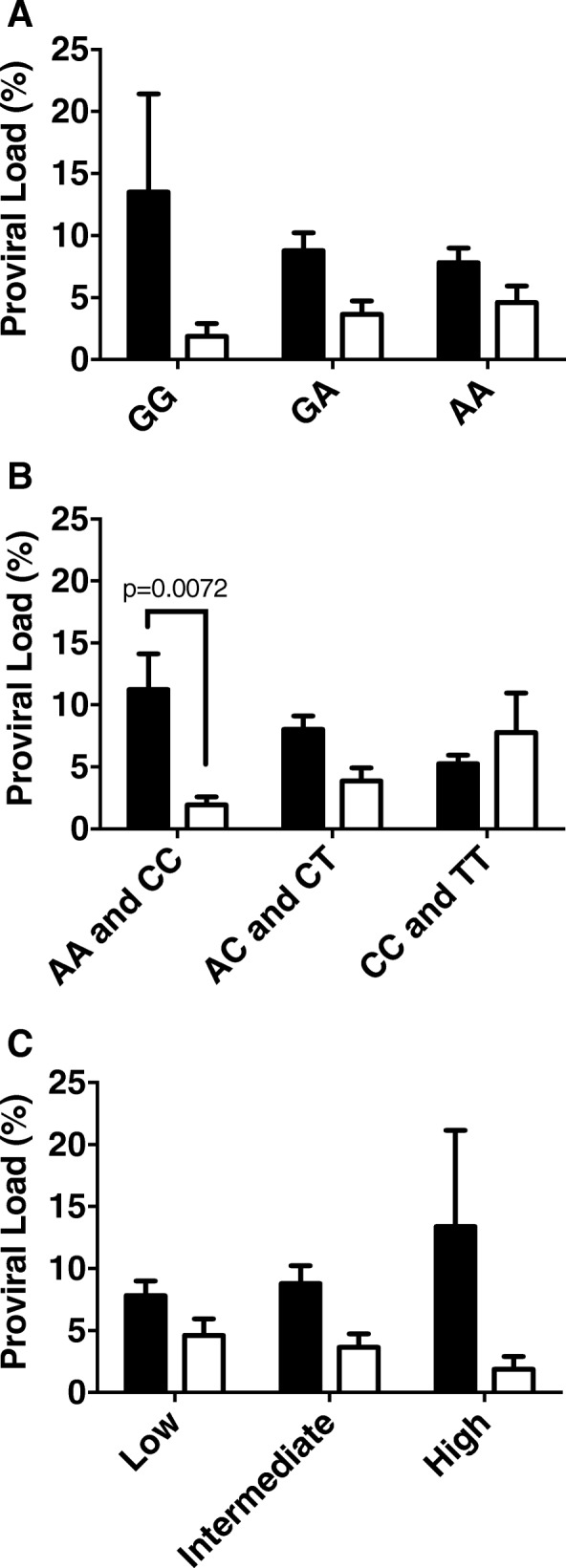
HTLV-1 pro viral load according with IL10 genotypes and phenotypes in HAM/TSP versus AC. The proviral load was expressed as percentage of infected blood leukocytes. **a** and **b** represent *IL10–1082G/A* and *IL-10 -592A/C* and *-819C/T* genotypes. **c** represents IL-10 phenotype. Statistical significances are indicated according with p value

Besides the PVL was higher in HAM/TSP than in AC, we found an association in *TNFA-308GG* (low producer), *IL6GG*/*GC* genotypes (high producer) and high TGF-β phenotype carriers between the HAM/TSP and the AC groups (Fig. 1). *IL10-592CC* and *IL10–819CC* (*p* = 0.0072) genotypes were associated with high PVL in HAM/TSP patients compared with the AC group, as shown in Fig. 2. No further association was identified between the different genotypes or phenotypes when the HAM/TSP or AC patients were analyzed. These results point to a lack of association between the PVL and these cytokine polymorphisms in our set of HTLV-1-infected Brazilian patients.

## Discussion

HAM/TSP could be the consequence of an inflammatory response of the host immune system; however, the majority of HTLV-1-infected individuals remain asymptomatic, indicating that HTLV-1 infection by itself is not sufficient to induce HAM/TSP. Neurons and glial cells are damaged by toxic or inflammatory products released from HTLV-1-infected T cells and the bystander damage by activated cytotoxic lymphocytes in the central nervous system [27]. Cytokines production varies among individuals, due in part to genetic factors and, in particular, the presence of polymorphisms in important regulatory regions, such as promoter regions. In this study, we aimed to establish an association between polymorphisms in cytokine-related genes (*TNFA, IL6, IFNG, TGFB* and *IL10*) and disease outcome. Our results demonstrated that there was no association between *TNFA-308G/A*, *IL6-174G/C*, *IFNG + 874 T/A*, *TGFB* at codons *+ 10 T/C* and *+ 25G/C*, *and IL10-1082G/A, -819C/T* and *-592C/A* polymorphisms and development of HAM/TSP in our set of the Brazilian population. To the best of our knowledge, few studies addressed information on cytokine SNPs and HTLV-1 infection in the Brazilian population.

In vitro studies have shown that HTLV-1 infected lymphocytes transpose the blood-brain barrier, changing its permeability by secretion of IL-1α and TNF-α, which increases the migration of lymphocytes through this site [28]. This migration could enhance the inflammatory response in the nervous system, causing damage in neural cells in vivo. Polymorphism in the promoter region of the *TNFA-308G/A* was described by Wilson et al. 1992 [29], where the replacement of the allele guanine *TNFA (−308G* or *TNF1*) for adenine in the *TNFA* allele *(−308A* or *TNF2*) has been associated with high production of this cytokine in European populations [29]. The *TNFA-308G/A* polymorphism was analyzed in adult T-cell leukemia/lymphoma versus AC in Japanese population, and no association between this SNP and disease outcome was found [20]. In our set of individuals typed for *TNFA-308G/A* polymorphism, we also did not find an association between this SNP and the HAM/TSP or AC groups. However, an association between the genotype *TNFA-308GG* (low producer) and high PVL was observed. Starling et al. 2013 showed an inverse correlation between TNF-α production and PVL in HAM/TSP patients [30], corroborating our results and indicating a possible protective role of TNF-α in the control of pro viral load. Our data suggest a possible effect of *TNFA-308GG* genotype in PVL not related to disease outcome.

The *IL6 -174G/C* is associated with changes in cytokine production, where G allele carriers show higher production of IL-6 compared to C allele carriers [11]. Gadelha and colleagues [21] described no association between HAM/TSP and AC in *IL6 -174G/C* in a Northeast Brazilian population, although this SNP was a risk factor for HAM/TSP when compared with oligosymptomatic patients. Although none of the *IL-6* genetic background was associated with HAM/TSP outcome, we observed an increased level of PVL in HAM/TSP patients carrying the higher IL-6 producer (*IL6-174GG and CC*) genotypes, indicating that a high level of this inflammatory cytokine could be related to a worse scenario in HAM/TSP patients. In addition, IL-6 and TGF-β may induce the differentiation of a Th17 cell profile and increase central nervous system inflammation [31].

In HAM/TSP patients, there is a predominance of Th1 cytokines such as IFN-γ, reduction in Th2 cytokines such as IL-4 and IL-10, and increased production of neurotoxic cytokines affecting regions along the spinal cord [32]. The IFN-γ serum concentration is higher in HAM/TSP individuals than in asymptomatic individuals [6, 30]. Located in the first intron of the *IFNG* gene, the SNP + 874 *T/A* contributes to IFN-γ production, with T allele carriers presenting higher production of IFN-γ compared to A allele carriers [8]. Recently, Queiroz et al. 2018 showed higher prevalence of the *IFNG + 874 T/A* T allele among AC compared to HTLV-1 symptomatic carriers, although no differences in genotypes distribution were detected [20]. Queiroz et al. data agree with our results regard genotype distribution and PVL, differently of allelic distribution, where we did not find differences. This discordance might be due to different study approaches. Queiroz et al. enrolled in their study, HTLV-1 symptomatic carriers with diverse clinical manifestations, such as rheumatism, condition absent in our study. In addition, the patients of that study belonged to North region of Brazil, where differences in migration history and genetics background might occur compared to our study. Our results, in agreement with a previous study [33], demonstrated no significant differences for allele and genotype frequencies of the *IFNG* + 874 *T/A* SNP between HAM and AC. In our set of the Brazilian population, there was no statistical influence on any genotypes of *IFNG + 874 T/A* and PVL in the HAM/TSP or AC groups. However, our results were in discordance with those published by Rocha-Junior et al. [33], who reported a significant association between PVL and the *IFNG + 874AA* genotype, where this SNP was associated with low PVL. In addition to the two populations belonging to Brazil, the study also had a group included by Rocha-Junior that was from São Paulo State, where high Asiatic frequency is present; our study groups did not include any Asiatic individuals.

It has been shown that TGF- β signaling is critical for Foxp3 expression and T regulatory cells functions in HAM/TSP patients [34]. HAM/TSP patients showed low levels of TGF-β receptor II (TGF-βRII) and Smad7 (a TGF-β–inducible gene) in CD4+ T cells, when compared to healthy donors. In addition, TGF-βRII expression was inversely correlated with the HTLV-1 PVL. This evidence suggests that HTLV-1 can modulate the immune tolerance affecting both regulatory and effector T cells and contributing to the pathogenesis of HAM/TSP [5]. HTLV-1 infection in patients with Sjogren’s Syndrome led to an enhanced serum level of TGF-β and may be important for increased HTLV PVL [35]. The haplotype TG from the SNPs of *TGFB* at the codons *+ 10 T/C* and *+ 25G/C* has been associated with high production of this cytokine [13]. We did not observe association with these SNPs and HAM/TSP development. However, in our study, we observed higher PVL in HAM/TSP patients TGF-β high producers (+10TT or TC, and + 25GG) compared to AC, suggesting a role of this cytokine in control of the viral replication and disease prognosis. Additional factors might affect the expression of TGF-β during the HTLV-1 infection, changing the gene expression levels regardless the polymorphisms influence [5, 36, 37]. To the best of our knowledge, there is no previous work describing the *TGFB* SNP in HAM/TSP patients.

HTLV-1 infection in HAM/TSP patients does not alter the expression of IL-10 when compared with AC patients [38]. SNPs in the *IL10* gene are controversially associated with the HAM/TSP outcome. A Japanese population showed an association between the *IL10 -592A* allele and a protective effect, reducing risk of HAM/TSP [39]; however, this SNP was considered to be a risk factor for developing HTLV-1 infection and disease in an Iranian population [22]. No association between *IL10* SNPs and HAM/TSP outcome was observed in a Brazilian population [21] in a high-risk HTLV-1 prevalence area with 9.4/1000 habitants [40]. In our set of Brazilian population enrolled from Rio de Janeiro City, an intermediate HTLV-1 prevalence risk area, with 4.7/1000 habitants [40] there was no association with the disease outcome. At position *IL10–1082A/G,* an Iranian population showed no association with HAM/TSP in a co-dominant genetic model [22]. There were no previous reports describing the SNP in this position in Brazilian HTLV-1-infected individuals. Our results showed a predominance of intermediate producers of *IL10-1082AG* and *GG* genotypes among the HAM/TSP individuals (OR 2.42 [1.2–4.97] *p* = 0.01), predisposing to disease outcome, although the data lost significance after Bonferroni correction for multiple comparisons. Low-producer haplotypes (*IL10-1082A, − 819 T* and *-592A*) have been previously associated with AC or HAM/TSP compared with healthy controls [40]; however, no association was described between AC and HAM/TSP groups, as described in this study. Our results demonstrated that only *IL10–819CC* and *-592AA* carriers, presenting high levels of IL10 production, have the highest PVL when the HAM/TSP group was compared with the AC group.

The infection caused by HTLV-1 elicits a robust immune response with many factors affecting the cytokines gene expression, such as genetic population’s background, virus subtypes, immunomodulation and individual health status. Indeed, it has been shown that different subtypes of HTLV-1 are region restricted, besides the genetic background of ethnicities across the world [41]. This fact could explain association between polymorphisms of cytokine genes, such as *IL10 -592C/A* and *IL10–1082A/G,* and HTLV-1 infection in some specific populations. On the other hand, some polymorphisms, such as *TNF -308G/A, IFNG + 874 T/A, IL6 -174G/C*, are repeatedly not associated with the disease, regardless genetic background population, suggesting irrelevant role in the HTLV-1 infection. However, more studies in different populations are needed to confirm this hypothesis. Despite no association with disease development, some of these polymorphisms in cytokine genes, such as *IFNG + 874 T/A, TNF -308G/A, IL6 -174G/C, TGFB + 10 T/C and + 25G/C, IL10–819C/T* and *-592C/A* might change PVL, indicating a role of this polymorphisms in the controlling of the viral replication and disease prognosis.

Our study might be analyzed considering its limitations such as the number of patients enrolled and the unavailability to measure cytokines concentrations in plasma or cultured cells in the patients. As we used convenience samples, we had limitations regard sample size and biological material to perform the analysis. Despite these polymorphisms are well established to modulate the cytokines production, is not clear its role on the HTLV-1 infection. Future studies addressing the role of these polymorphisms in different populations, associated with the cytokines production, might clarify the role of these functional SNPs in the HTLV-1 infection.

## Conclusion

In conclusion, besides the importance given to pro- and anti-inflammatory cytokines in the outcome of HAM/TSP, we could not affirm that *TNFA-308G/A, IL6-174G/C, IFNG + 874 T/A, TGFB* at the codons *+ 10 T/C and* + *25G/C*, *IL10-592C/A and -819C/T, and -1082A/G* polymorphisms are related to either disease progression, even those related with the amount of secreted cytokines. Genetic background may be studied in other sets of populations to determine and understand the complex role of cytokine networks in HTLV-1 infection and improve the clinical studies for this disease.

## Abbreviations

AC: Asymptomatic carriers
HAM/TSP: HTLV-1-associated myelopathy/tropical spastic paraparesis
HTLV-1: Human T-lymphotropic virus 1
PVL: Proviral load
SNP: Single nucleotide polymorphism
